# Nesting success of red-winged blackbirds (*Agelaius phoeniceus*) in marshes in an anthropogenic landscape

**DOI:** 10.1098/rsos.220266

**Published:** 2022-07-27

**Authors:** Scott K. Robinson, Holly M. McChesney

**Affiliations:** ^1^ Florida Museum of Natural History, University of Florida, Gainesville, FL 32611, USA; ^2^ Arcadis U.S. Inc., Wexford, PA 15090, USA

**Keywords:** reproductive success, polychlorinated biphenyls, red-winged blackbirds

## Abstract

Recent analyses show significant population declines in many abundant avian species, especially marsh-nesting species including the red-winged blackbird (RWBL). Hypothesized causes include reduced nesting success resulting from changing land-use patterns and exposure to contaminants. Our goal was to test the hypothesis that landscape and nest characteristics as well as exposure to polychlorinated biphenyls (PCBs) correlate with nesting success. From 2008 to 2014, we measured clutch size, egg and nestling mass, hatching and fledging success and daily survival of 1293 RWBL nests from 32 marshes in the Hudson River valley of New York. Using generalized linear effect and survival models, we found that: (i) Julian date was negatively related to hatching success and clutch size but positively related to egg mass; (ii) nest height was negatively related to hatching success; (iii) nestling mass decreased with increased nest density and distance to edges; (iv) fledging success was significantly lower in nests closer to the ground that were far from water; and (v) clutch size and daily survival were higher in nests farther from water. Results showed that nesting success was correlated with variables associated with flooding, population density and predation and provided no support for the predicted negative effects of PCB exposure.

## 1. Introduction

A recent meta-analysis of population trends for the last 50 years in North America has drawn attention to large-scale population declines in species that are or were until recently abundant and widespread in human-altered landscapes [1]. These declines have been severe in the American blackbirds (Icteridae), especially those that nest in marshes [1]. For example, across the United States, red-winged blackbird (RWBL) (*Agelaius phoeniceus*) populations have declined 0.8% per year from 1966 to 2019, with more precipitous declines in localized regions. In New York State, RWBL populations have declined by 1.8% per year from 1966 to 2019 [2]. One hypothesized cause of these declines is that conditions in human-dominated landscapes may be changing in ways that reduce nesting habitat quality [3–5]. Declines have been attributed to decreasing nesting productivity resulting from increasing exposure to environmental toxins and contaminants [6–8] including polychlorinated biphenyls (PCBs) [9], habitat loss and fragmentation [10,11], agricultural intensification [12–14], urbanization [15], increasing predator populations [10,16,17] and invasive species [4,18,19]. To understand possible drivers of these population declines it is necessary to identify the factors that affect nesting success in complex, human-dominated landscapes where the steepest declines have occurred [1].

Wetland habitats may be especially sensitive to human-induced changes in the environment [20–22]. Most wetlands that persist in human-dominated landscapes are small, edge-dominated patches in areas that are too low to drain or along ponds and drainage ditches used to manage stormwater [20–25]. These wetland fragments are surrounded by crops, pastures, human habitations and roads, all of which can negatively affect nesting success [21,26–29] (but see [11] for wetland birds). Additionally, environmental contaminants tend to accumulate in wetlands [22,30,31] creating concern for wildlife that reside in contaminated wetlands and marshes, especially those species at the top of food webs [32,33].

Factors that reduce nesting success in marsh habitat may have particularly severe consequences for the American blackbirds (Icteridae) that nest at high population densities, or even colonially, in marshes [34,35]. RWBL, tricolored (*A. tricolor*) and yellow-headed (*Xanthocephalus xanthocephalus*) blackbirds, all of which are declining range-wide, show strong preferences for nesting in marshes throughout their breeding ranges [27,34–36]. Marshes contain rich insect resources [35,37], especially emerging aquatic insects, that provide breeding birds with abundant, high-quality food resources that are rich in protein and long-chain polyunsaturated fatty acids [9,35,37–40]. Marshes are also seldom mowed during the breeding season, a problem for birds nesting in upland fields [36]. Nesting in marshes may also provide protection against predators and brood parasites [41,42], an effect that may be enhanced by improved group defence in marshes where blackbirds nest at high population densities [43–46]. Population reductions within marshes may further erode these defences if there are not enough individuals to chase away predators and parasites. Vierling [29] argued that natural wetlands act as source habitats that may be helping to sustain regional populations of RWBL in an urbanizing landscape. Any factors that reduce nesting success within remaining marshes, therefore, may contribute disproportionately to regional population declines [21,29,36,47].

A primary goal of this study was to determine if the nesting success of marsh-nesting RWBLs in areas heavily contaminated with PCBs differed from areas where there was no exposure to PCBs. PCBs were released from a nearby Superfund site, designated by the United States Environmental Protection Agency as requiring a long-term action to remediate hazardous contamination [48]. PCB concentrations in the soils and sediments of the investigated portions of the Hudson River and its immediate floodplain are among the highest ever recorded, prompting numerous studies on the effects of PCBs on multiple biota [49,50], including several species of birds [9,51–53]. PCB concentrations in RWBL eggs collected along the upper Hudson River ranged from 0.08 to 34.8 mg kg^−1^ [54] (see electronic supplementary material, figure S1) and include concentrations with potential for negative effects on nesting success. Results from studies of nesting success of birds in relation to exposure to PCBs have been mixed, with some studies reporting reductions in nesting success [9,55] and others showing no effects [51,52,56]. Effects of PCBs on nesting success are more prevalent in laboratory studies where exposure to PCBs may exceed potential exposure in the wild [57]. The extent of potential effects ranges from altered parental care that can affect hatch rates to complete embryo death [57–59]. Results from field studies are more variable even within single studies. For example, significantly reduced hatchability and increased nest abandonment associated with PCBs in tree swallow nests located along the upper Hudson River varied by year [9]; whereas Custer *et al*. [55] reported consistent reductions in tree swallow hatching success relative to PCB concentration in eggs.

Birds accumulate PCBs through their diet, especially via the consumption of invertebrates. Marsh-nesting blackbirds may be especially prone to contaminants in sediments because they eat emerging odonates that are near the top of aquatic food webs [35,37,39]. RWBLs, however, are considered to be only moderately exposed to contaminants in the sediment [60] because they feed both in marshes, where exposure to contaminated sediment may be high, and in the uplands surrounding marshes, where exposure to sediment is limited [60–62].

This study also was intended to test potential effects of PCBs on nesting success relative to other factors that have been shown to affect RWBL nesting success. These factors include within-season timing of nesting [63,64], proximity to wooded edges and buildings [21,65], but see [29,47,66], nest height [42] and nest density and distribution within marshes [42–45,67,68]. We predicted that nest predation rates would decrease with proximity to open water (a proxy for water depth) [65] and with nest density (increased group defence) [45] and would increase with proximity to human habitations or forest edges [29,47,69]. Increasing nest height should also provide protection against flooding [42]. We further predicted that measures of nesting success that control for effects of nest predation and flooding (e.g. clutch size, egg mass, hatching success, nestling mass and fledging success) would decrease with Julian date [70], nest density (density-dependent reductions in food supply) [42], and with increasing exposure to PCBs.

## 2. Material and methods

### 2.1. Study area

**Figure 1.**
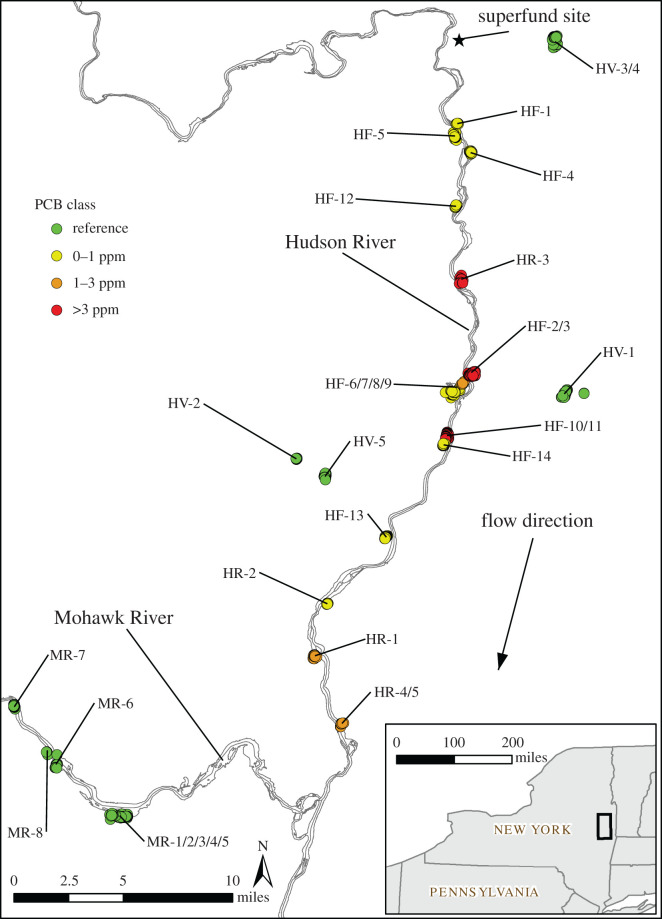
Marshes investigated during six field-seasons in 2008–2014 along the upper Hudson River and Mohawk River. Marshes divided into PCB categories based on geographical proximity to measured PCB concentrations in soil or sediment.

**Figure 2.**
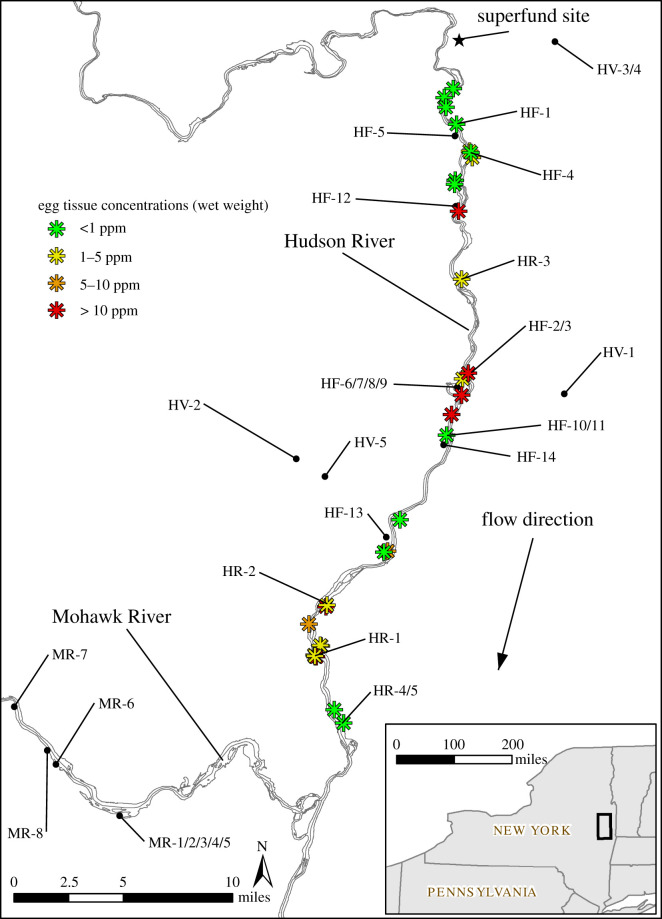
Marshes investigated during six field-seasons in 2008–2014 along the upper Hudson River and Mohawk River shown in relation to locations of known RWBL egg PCB concentrations.

This study was conducted at 32 marsh (primarily cattail (Typha)-dominated) study sites during six field seasons, 2008–2014 (figure 1; electronic supplementary material, figures S2–S24 showing maps of all marshes). Each marsh covers a distinct contiguous area, of which many appear to be fragments of historical marsh areas. The investigated marshes ranged in size from 0.12 to 8.1 ha (table 1). PCB exposure from a source of contamination on the upper Hudson River has been reported and may have the potential to affect the nesting productivity of RWBL. In 2002, 40 red-winged blackbird eggs from nests found within the upper Hudson River floodplain were collected and analysed for PCBs [54]. Sampling results show that PCB concentrations in eggs ranged from 0.08 to 34.8 ppm (figure 2), with the lowest average concentration found south of the Mohawk River [54]. For this reason, marshes situated within four distinct regions were chosen to represent different levels of exposure to waters and sediments contaminated with PCBs (table 1). (i) Hudson River Region: six of the marshes were directly connected to the Hudson River where they were exposed throughout the year to sediments contaminated with PCBs from the Superfund site on the upper Hudson River. (ii) Mohawk River Region: eight additional marshes were selected from the nearby Mohawk River where nests were also exposed directly to river sediments, but not to PCBs from the Superfund site, which was on the upper Hudson River. A series of dams on the two rivers prevent any mixing of water from the two rivers in the study area. The nearest known PCB source in the Mohawk River is over 100 miles upstream from where it flows into the Hudson River [71]. PCBs were not detected in most water column and sediment samples collected from this portion of the Mohawk River suggesting that nest data collected from nearby marshes could represent reference conditions [71–73] . The Mohawk River Region was used as the reference area for the nests located directly on the border of the Hudson River because there were no significant marshes located on the Hudson River upstream of the source of PCBs. (iii) Hudson Floodplain Region: 13 marshes in the floodplain of the upper Hudson River were chosen because they were exposed to Hudson River sediment deposition during spring flooding events. These sites ranged from 6 to 41 km along the river downstream from the Superfund site. (iv) Hudson Valley Region: five additional marshes were selected in the Hudson valley that were above the 100-year floodplain elevation such that they were not exposed to PCBs from the Superfund site. These latter marshes were considered as controls for the Hudson Floodplain marshes. Within each of these regions, marshes were selected at random from those identified by satellite images and topographic maps showing floodplain limits, with the additional constraints that they had to be accessible and have landowners' permission to conduct research on their property.

**Table 1.** List of marshes investigated during six field-seasons in 2008–2014 in various regions along the upper Hudson River and Mohawk River with their corresponding area (in hectares) and surrounding land use.

region	marsh ID	marsh area/area surved (ha)**^a^**	surrounding land use
Hudson Floodplain	HF-1	0.98	pasture and grassland
	HF-2	3.67	row-crop
	HF-3	2.13	row-crop
	HF-4	2.37	semi-urban/residential
	HF-5	6.33	row-crop
	HF-6	1.90	row-crop
	HF-7	0.40	row-crop
	HF-8	2.46	row-crop
	HF-9	1.68	row-crop
	HF-10	2.23	row-crop
	HF-11	0.27	row-crop
	HF-12	1.38	pasture and grassland
	HF-13	1.49	semi-urban/residential
	HF-14	1.00	semi-urban/residential
Hudson River	HR-1	2.09	semi-urban/residential
	HR-2	0.67	semi-urban/residential
	HR-3	0.98	semi-urban/residential
	HR-4	0.36	semi-urban/residential
	HR-5	0.12	semi-urban/residential
Hudson Valley	HV-1	5.39-8.13	pasture and grassland
	HV-2	0.66	semi-urban/residential
	HV-3	1.08-2.49	pasture and grassland
	HV-4	4.03	pasture and grassland
	HV-5	0.65-4.28	pasture and grassland
Mohawk	MR-1	1.11	forest
	MR-2	0.24	forest
	MR-3	0.88	forest
	MR-4	0.77	forest
	MR-5	4.77-7.74	forest
	MR-6	0.55	forest
	MR-7	1.92	semi-urban/residential
	MR-8	1.11	semi-urban/residential

^a^Due to safety, field staff and accessibility considerations, survey areas were limited in some years. Not all marshes were surveyed each year.

Marshes were all located in landscapes dominated by intensive human land uses such as row-crops and pastures or hayfields, roads and yards surrounding buildings (table 1, electronic supplementary material, figures S1–S23). Some were bordered by floodplain forest or rows of trees and shrubs growing at the edges of the marshes, which formed distinct edges. Other marshes, especially those along the rivers also had distinct edges bordering open water that was too deep to sustain emergent aquatic vegetation such as cattails. Marshes bordering agricultural fields, pastures and hayfields did not form distinct, permanent edges because water levels varied greatly throughout the study causing marshes to expand and contract. For this reason, we restricted our analyses of distances to edges to woody borders and to distances to buildings as an indicator of residential effects on a marsh [29,47]. Nests were mostly in cattails (*Typha* spp.; 74%), although a few were in other plants such as common buttonbush (*Cephalanthus occidentalis*; 7.7%), sedge (*Carex* spp.; 4.4%), invasive purple loosestrife (*Lythrum salicaria*; 3.9%) and other vegetation types.

### 2.2. Field methods

We used the methods described in Martin & Geupel [74] to monitor nests and estimate hatching and fledging success. Most marshes were searched each year, with the extent of the surveys dependent primarily on accessibility, safety concerns and field staff. Marshes were searched along transects that were roughly 5 m apart. Two observers walked slowly along these transects searching for all active nests. This method was designed to find all nests that were active on the day of searching. Each marsh was searched twice—once early in the season in May and once late in the season in June. Nest searching was only conducted during days with no precipitation and temperatures greater than 10°C to minimize losses caused by interference from investigators. Because we were studying many marshes spread over a large area (figure 1), we were only able to visit nests every 3–4 days, which introduced some possible error in our estimates of hatching and fledging success [74].

When nests were found, we recorded the nest height above the ground or the water surface and the plant species in which it was located. Each nest was mapped using GPS (stabilized readings accuracy +/− 3 m). Nests were flagged 2–3 m from the nest location to avoid attracting the attention of predators [75]. Each time a nest was visited, we recorded its contents. We tried to weigh the eggs as early in the incubation period as possible given the 3–4 day interval between visits. If the nest appeared to have a complete clutch (3–4 eggs; see below), we weighed the eggs on the first visit and only weighed them again if an additional egg appeared. If the nest was found empty or with a smaller clutch (1–2 eggs), we waited until the next visit to weigh the eggs. Hatching success was estimated from the number of newly hatched nestlings and the presence of unhatched eggs in nests at least 1–2 days after hatching based on the feather development of the nestlings. We did not use nests found during the nestling stage to estimate hatching success. If eggs disappeared during incubation prior to hatching, we did not count these as unhatched eggs as they could have represented partial predation or brood reduction by the parent.

Nestlings were weighed and measured (tarsus length and wing cord length) and their feather development described. We tried to measure the nestlings as late in the nestling period as possible but before they were sufficiently developed that they would fledge when we approached (6–9 days after hatching). Based on nests with known hatching dates, we knew that flight feather development (sheathed, partially sheathed, unsheathed) was a reliable indicator that nestlings were 6–9 days post-hatching (unpublished data).

Fledging success estimates were based on the proportion of nests in which young survived long enough to fledge and there was no evidence of predation in the late nestling period. If nests were empty on the last check and they were far enough along in development to possibly have fledged, we searched the area around the nest for fledglings and for females carrying food [71]. If we did not find any evidence of fledglings, we then checked the nest for signs of likely fledging, such as a build-up of faeces in the nest [71]. If nests were damaged, we considered this as evidence of predation.

We used the standards proposed by the Ornithological Council [76,77] regarding the humane treatment of birds. The only animal contact in this study occurred when we weighed and measured nestlings, a procedure that resulted in no harm to the individuals and lasted less than 5 min per nestling. All work was done under permits from NYSDEC (LCP #1457) and US Bird Banding Laboratory (#23373).

### 2.3. Predictor variables

Predictor variables included specific nest characteristics, marsh habitat characteristics and estimated PCB exposure, all of which we had hypothesized to affect nesting success. Nest characteristics included nest height, date the nest was found and dates both egg and hatchling mass were collected. Marsh habitat characteristics included distance to woody edge, nest density and distance to open water and buildings. Distance measures were derived using spatial analyses. Collected GPS coordinates for each nest were compared with aerial photos and existing geographical information system (GIS) databases that included land use, hydrology, roads and wetlands. Distance to edge was calculated as the distance from each nest to the nearest woodland or human structure. Distance to open water was calculated as the distance from each nest to the closest unvegetated open water of ponds or rivers.

We used nesting density as a proxy for population density of RWBLs in marshes. We derived nest density for each marsh using a time-to-event nest abundance estimator that accounts for variability in detection and survival probability based on site (marsh) and nesting stage (incubation or nestling) [78,79]. Based on published studies, the average duration of the incubation period was assumed to be 11 days [80]. Models used to derive annual abundance estimates for each marsh were calculated for each survey year using the nestAbund package in program R [78]. Final models were chosen based on Akaike's information criteria (AIC) and varied by year. Annual abundance estimates for each marsh were used in conjunction with the estimated marsh areas to provide annual nest density estimates for each marsh. When a marsh was not able to be searched in its entirety in a given year, annual density estimates were adjusted to account for the reduction in area searched (electronic supplementary material, table S1).

PCB exposure categories were defined by the maximum sediment/soil total PCB concentration obtained from existing samples collected within the contiguous marsh areas located in the upper Hudson River floodplains. When no existing samples were present, the maximum concentration of the nearest existing soil sample was used. The upper Hudson River floodplains have been heavily sampled for PCBs. When a sampling location was not present within a given marsh, nearby samples were available within an average distance of 79 m (range: 0.6 to 279 m, standard deviation: 78 m). Marshes from the Mohawk River and Hudson Valley Regions were located more than 2300 m from PCB sampling locations and were considered reference locations. Given the uncertainty in associated PCB concentrations resulting from several marshes without collocated PCB samples, PCB data is treated as an index of exposure using an ordinal variable based on nest PCB categories of: (i) reference; (ii) 0–1 ppm PCB; (iii) 1–3 ppm PCB; and (iv) greater than 3 ppm PCB, corresponding to a range of potential PCB effects from no effect to high potential effect (figure 1). Measured PCB concentrations from eggs collected within the Hudson River floodplain [81] correspond somewhat with the PCB categories derived for nearby marshes included in this study but show no obvious trend with distance from the PCB source (figure 2 and electronic supplementary material, figure S1).

### 2.4. Statistical methods

We used generalized linear mixed-effects models (function glmer in the R package lme4) [82] and generalized linear models (function glm in the R package stats) [83] to assess relationships between environmental and habitat variables and five reproductive metrics: (i) clutch size, (ii) egg mass, (iii) hatching success (proportion of eggs that hatched, weighted by the total clutch size), (iv) nestling mass, and (v) fledging success (proportion of nestlings that fledged, weighted by the total brood size). Each of these five non-independent response variables was analysed separately, so we adjusted the alpha value to 0.01. Clutch size was not highly variable (1190 of 1300 clutches had either three or four eggs), so we treated it as a bivariate factor with clutches of up to three eggs scored as a 0 and clutches of four or more eggs scored as a 1. We used a binomial distribution to analyse clutch size as well as hatching and fledging success for each individual egg and nestling, and a Gaussian distribution for egg and nestling mass. Egg mass was squared prior to analysis to better fit a Gaussian distribution. We assessed fit of models with Gaussian distributions by visual inspection of plots of model residuals versus fitted values, sample versus theoretical quantiles (q-q plot), and frequency distributions of model residuals, as well as plots of model residuals against each fixed effect. Similar evaluations are not effective for assessment of binomial models, but we balanced all predictors through transformations and scaling to improve model fit.

We started with similar global models for all response variables, which included variables that accounted for effects of seasonality or timing (Julian day of year for onset of incubation or number of days since the onset of incubation), water depth (distance to open water), flooding (distance to open water and nest height), predation (nest density, distance to open water and nest height), and PCB exposure (nest PCB category). Initial analyses of hatching success, fledging success and clutch size included year, treated as a categorical factor, as a random intercept to control for unmeasured among-year variation in environmental conditions that might influence reproductive success. Year was retained in the hatching and fledging success models, where intra-class correlation (ICC) suggested that observations within Year could be correlated. Analyses of egg mass and nestling mass included nest ID as a random intercept, to account for non-independence of measurements of eggs and offspring from the same nest. All initial analyses included marsh region (Hudson Floodplain, Hudson River, Hudson Valley and Mohawk) to account for unmeasured regional variation that might influence reproductive success. Region was retained in the hatching and fledging success models based on ICC results suggesting correlation among results by region.

A series of 24 *a priori* models was developed for each response variable to account for potential interactions (table 2). We treated PCB level as ordinal data for analyses, which facilitated consideration of the effects of PCBs that might depend on other environmental factors (i.e. inclusion in interaction effects). Distance to edge and distance to open water were right-skewed and so were log-transformed prior to analyses. Nest density had a slightly right-skewed distribution that was better improved through square-root transformation. We scaled all continuous fixed effects (after any required transformations) to a mean of zero and standard deviation of one, which allows for more direct comparison of effect sizes among fixed effects. Results were plotted with unscaled data, for ease of interpretation. Multi-collinearity among continuous fixed effects was not an issue (all pairwise |*r*| < 0.31).

**Table 2.** List of fixed variables included in 24 *a priori* models used to evaluate nesting success response data collected from marshes in 2008–2014 in various regions along the upper Hudson River and Mohawk River. Random variables were also included in each model to offset unmeasured variation among-years and by region and to account for non-independence of eggs and offspring from the same nests.

model	fixed variables only
1. null	.
2. global	DOY + (dWater × NestHt) + (dWater × PCB) + (Density × dEdge)
3. day of year	DOY
4. PCB	PCB
5. diet	dWater
6. flooding^a^	dWater × NestHt
7. predation^b^	NestHt + (Density × dEdge)
8. DOY + PCB	DOY + PCB
9. DOY + Diet	DOY + dWater
10. DOY + flooding^a^	DOY + (dWater × NestHt)
11. DOY + predation^b^	DOY + NestHt + (Density × dEdge)
12. DOY + flooding + predation^ab^	DOY + (dWater × NestHt) + (Density × dEdge)
13. DOY + diet + predation^b^	DOY + dWater + NestHt + (Density × dEdge)
14. PCB, diet interaction	PCB × dWater
15. DOY+ PCB, diet interaction	DOY + PCB + dWater
16. PCB + flooding^a^	PCB + (dWater × NestHt)
17. DOY + PCB + flooding^a^	DOY + PCB + (dWater × NestHt)
18. PCB + predation^b^	PCB + NestHt + (Density × dEdge)
19. DOY + PCB + predation^b^	DOY + PCB + NestHt + (Density × dEdge)
20. PCB, diet interaction + predation^b^	(PCB × dWater) + NestHt + (Density × dEdge)
21. DOY + PCB, diet interaction + predation^b^	DOY + (PCB × dWater) + NestHt + (Density × dEdge)
22. PCB + predation + flooding^ab^	PCB + (NestHt × dWater) + (Density × dEdge)
23. diet + predation^b^	dWater + NestHt + (Density × dEdge)
24. flooding + predation^ab^	(NestHt × dWater) + (Density × dEdge)
random variables^c^	**Response Variables**
year	proportion hatched, fledging success
region	proportion hatched, fledging success
nest ID^d^	nestling mass, egg mass

^a^Not included when modelling datasets excluded known flooded nests.

^b^Not included when modelling datasets excluded known predated nests.

^c^Not included in models where the intra-class correlation (ICC) suggests that observations within the random variables are not correlated.

^d^Used for egg and nestling mass models only. Model factors include Julian date of onset of incubation (DOY), distance to open water (dWater), nest height categorized as less than or equal to 0.5 m or greater than 0.5 m (NestHt), nest density (Density), distance to edge (dEdge), and PCB. For the egg mass and nestling mass models, additional fixed variables were included in all models, to account for age of the egg or nestling (incubation day and nestling age, respectively).

We included scaled calendar day of the onset of incubation as a covariate in all global models. RWBLs may produce multiple broods per year [80], but the expectation is that reproductive investment might decline across the breeding season (table 2). We included nest height as a covariate in analyses of hatching and fledging success because nest height might alter exposure to some sources of nest failure (e.g. flooding or predation). There was little variability in nest height for nests within 5 m of the surface and the majority of nests known to be flooded were built less than or equal to 0.5 m of the surface. Therefore, to best represent risk from flooding, we classified nest height as a categorical variable scored low for nests less than or equal to 0.5 m and high for nests greater than 0.5 m from the surface. Eggs were weighed at differing days across the approximately 14-day incubation period, and so we included the scaled day of incubation as a covariate to control for water loss, which reduces egg mass over the course of incubation. Lastly, because nestlings were weighed at different ages (range 5–12 days old), we included a scaled estimate of nestling age as a covariate in analyses of nestling mass. All covariates were included only as main effects and not considered in any interaction terms.

Initial analyses of hatching success, fledging success and clutch size included year, treated as a categorical factor, as a random intercept to control for unmeasured among-year variation in environmental conditions that might influence reproductive success. Inclusion of year improved model fit for all global models, as indicated by a lower Akaike's information criterion corrected for small sample size (AICc) for global models with versus without (using a generalized linear model with a binomial distribution) this random effect. Analyses of egg mass and nestling mass included nest ID as a random intercept, to account for non-independence of measurements of eggs and offspring from the same nest.

To calculate model-averaged parameter estimates (function model.ave in the R package MuMln) [84], we included model results for each nest success response variable that were ranked within 2 AICc of the top-ranked model (i.e. lowest AICc). Model-averaged results did not differ qualitatively from analyses that used the top-ranked model, although effects with weak support in the top-ranked model (i.e. 0.05 < *p* < 0.10) received less support in model-averaged results. Given the relatively large sample size for all analyses, these marginal effects are unlikely to be biologically important. We used the top-ranked model to generate plots to illustrate any effects identified as important following model averaging.

We used three different datasets for analyses of hatching and fledging success: one that included all nests, one that excluded nests that failed due to predation during incubation or the nestling stage (depending on the response variable being analysed), and a final one that excluded depredated nests as well as nests that failed due to flooding during the relevant breeding stage. Nests that failed during incubation were not included in analyses of fledging success. Predictor variables were adjusted to exclude those that corresponded directly with the excluded nests (table 2). For example, the fixed variables that corresponded with predation were not included when predated nests were removed from the dataset. Comparisons of results across these three complementary analyses provide insight into the influence of flooding and predation on associations between reproductive success and habitat characteristics (i.e. the fixed effects). We analysed the remaining response variables (clutch size, egg mass, nestling mass) using only one dataset, which included all data regardless of eventual nest outcome.

We evaluated daily nest survival as a function of the predictor variables using the RMark package [85]. RMark acts as an interface for the MARK program [86]. The same set of 24 *a priori* models evaluated for the generalized linear and mixed effect models were included in the analysis (table 2). Random variables were not included in the daily nest survival models; however, Year was included as a categorical variable for all models to account for annual variability due to weather conditions and other unmeasured among-year variability in environmental conditions. Nest age, the number of days since the onset of incubation, was also included as a continuous variable in all models to account for known variability in daily survival over the nesting period [87,88]. As with the previous models, distance to edge and distance to open water were log-transformed and nest density was square-root transformed prior to analysis.

Competing daily survival models were ranked based on AICc. Modelled coefficients of predictor variables included in models ranked within 2 AICc of the topped ranked model were considered to have strong support when their confidence intervals did not include zero [89]. Potential effects of highly supported predictor variables were evaluated based on the highest ranked model in which they were included using mean values of continuous variables and categorical variables that best represent mean daily survival from egg incubation through fledging.

## 3. Results

Reproductive metrics and nest characteristics used as fixed effects are summarized by region in tables 3 and 4, respectively. All relationships between reproductive metrics and fixed effects are reported with an effect estimate (*β*) ± standard error derived from full model averaging across all models ranked within 2 AICc of the top-ranked model (table 5). Effect estimates can be compared among continuous fixed effects within a model because they were scaled (mean = 0, s.d. = 1) prior to analysis.

**Table 3.** Summary of reproductive metrics for marshes investigated during six field-seasons from 2008 to 2014 in various regions along the upper Hudson and Mohawk Rivers.

region	egg mass (g)	proportion hatched	nestling mass (g)	proportion fledged	clutch size
	mean (s.e.)	mean (s.e.)	mean (s.e.)	mean (s.e.)	mean (s.e.)
Hudson River Floodplain	3.88 (0.014)	0.64 (0.018)	30.07 (0.246)	0.75 (0.02)	3.58 (0.028)
Hudson River	3.96 (0.027)	0.58 (0.035)	31.02 (0.515)	0.79 (0.037)	3.59 (0.053)
Hudson Valley	3.9 (0.016)	0.74 (0.021)	29.92 (0.255)	0.71 (0.024)	3.59 (0.037)
Mohawk River	3.93 (0.022)	0.51 (0.028)	29.62 (0.474)	0.72 (0.034)	3.41 (0.044)

**Table 4.** Summary of nest characteristics for marshes investigated during six field-seasons from 2008 to 2014 in various regions along the upper Hudson and Mohawk Rivers.

region	day of year	PCB class	distance to edge (m)	distance to water (m)	nest density
	mean (s.e.)	mean (s.e.)	mean (s.e.)	mean (s.e.)	mean (s.e.)
Hudson River Floodplain	143.3 (0.533)	2.9 (0.041)	67.65 (1.726)	71.54 (2.815)	9.04 (0.242)
Hudson River	144.99 (0.967)	3.1 (0.047)	60.02 (3.403)	17.45 (1.337)	9.86 (0.535)
Hudson Valley	141.61 (0.69)	1 (0.003)	42.92 (1.551)	34.66 (1.361)	13.69 (0.5)
Mohawk River	146.7 (0.848)	1.01 (0.008)	25.01 (1.036)	27.91 (1.433)	11.6 (0.565)

**Table 5.** Parameter estimates for fixed effects of models within 2 AICc of top-ranked models that compare nest success of red-winged blackbird with habitat and nest characteristics. The models listed were used to derive model averaged parameter estimates for fixed effects that best explain variation in nest success parameters. Data evaluated in the models were collected from marshes in 2008–2014 in various regions along the upper Hudson and Mohawk Rivers.

name	intercept	incubation day (egg mass only)	nestling age (nestling mass only)	nest density (no. ha ^−1^)	distance to edge (m)	distance to water (m)	day of year	nest height (high/low)	PCB category	interaction: density and distance to edge	interaction: distance to water and nest height	interaction: distance to water & PCB category	family	d.f.	logLik	AICc	delta	model likelihood	AICwt
clutch size (categorical; ≤3 versus ≥4)																			
	0.367	−	−	−	−	0.174	−0.721	−	−	−	−	−	binomial(logit)	3	−799.36	1604.74	0	1.00	1.00
egg mass (g)																			
	15.423	−0.992	−	−	−	−	0.336	−	−	−	−	−	Gaussian(identity)	5	−5218.86	10 447.74	0	1.00	1.00
number of hatchlings (all locations)																			
	0.645	−	−	−	−	0.092	−0.165	+	−	−	+	−	binomial(logit)	7	−2457.49	4929.06	0	1.00	0.40
	0.621	−	−	−0.027	−0.064	0.089	−0.165	+	−	−0.049	+		binomial(logit)	10	−2455.01	4930.20	1.14	0.57	0.23
	0.631	−	−	−0.027	−0.064	0.141	−0.164	+	−	−0.054	−	−	binomial(logit)	9	−2456.11	4930.36	1.30	0.52	0.21
	0.640	−	−	−	−	0.089	−0.164	+	−0.025	−	+	−	binomial(logit)	8	−2457.39	4930.89	1.83	0.40	0.16
number of hatchlings (no predated nests)																			
	1.699	−	−	−	−	0.024	–	+	−	−	+	−	binomial(logit)	6	−1257.92	2527.93	0	1.00	0.44
	1.700	−	−	−	−	0.021	−0.066	+	−	−	+	−	binomial(logit)	7	−1257.02	2528.16	0.23	0.89	0.39
	1.703	−	−	−	−	0.027	−	+	0.023	−	+	−	binomial(logit)	7	−1257.88	2529.88	1.95	0.38	0.17
number of hatchlings (no predated/flooded nests)																			
	1.692	−		−	−	−	−0.080	−	−	−	−	−	binomial(logit)	4	−1028.56	2065.16	0	1.00	0.42
	1.700	−		–	–	0.042	−0.079	–	–	–	–	–	binomial(logit)	5	−1028.23	2066.52	1.36	0.51	0.21
	1.695	–		−	−	−	−0.082	–	0.046	−	−	−	binomial(logit)	5	−1028.38	2066.83	1.67	0.43	0.18
	1.703	−		−	−	0.045	−	−	−	−	−	−	binomial(logit)	4	−1029.40	2066.85	1.69	0.43	0.18
nestling mass (g)																			
	29.964	–	1.918	−0.473	−0.496	−	−	+	0.503	−0.253	−	−	Gaussian(identity)	9	−4539.14	9096.40	0	1.00	0.72
	29.954	−	1.924	−0.468	−0.498	−	0.071	+	0.504	−0.253	−	−	Gaussian(identity)	10	−4539.05	9098.26	1.86	0.40	0.28
fledging success (all locations)																			
	1.021	−		0.117	−0.048	0.070	–	+	−	0.042	+	−	binomial(logit)	9	−1454.09	2926.38	0	1.00	0.44
	1.009	−	−	0.118	−0.043	0.057	−	+	−0.097	0.041	+	−	binomial(logit)	10	−1453.50	2927.25	0.87	0.65	0.28
	1.020	−	−	0.119	−0.047	0.072	0.052	+	–	0.042	+	−	binomial(logit)	10	−1453.51	2927.26	0.88	0.64	0.28
fledging success (no predated nests)																			
	1.988	−	−	−	−	−	−	−	−	−	−	−	binomial(logit)	3	−725.82	1457.67	0	1.00	0.44
	1.980	−	−	−	−	−0.046	−	−	−	−	−	−	binomial(logit)	4	−725.58	1459.22	1.55	0.46	0.20
	1.989	−	−	−	−	−	−	−	−0.054	−	−	−	binomial(logit)	4	−725.71	1459.47	1.81	0.40	0.18
	1.990	−	−	−	−	−	0.023	−	−	−	−	−	binomial(logit)	4	−725.76	1459.57	1.91	0.39	0.17
fledging Success (no predated/flooded nests)																			
	2.139	–	–	–	–	–	–	–	–	–	–	–	binomial(logit)	3	−665.43	1336.89	0	1.00	0.44
	2.124	–	–	–	–	−0.095	–	–	–	–	–	–	binomial(logit)	4	−664.51	1337.07	0.18	0.92	0.40
	2.140	–	–	–	–	–	–	−0.029	–	–	–	–	binomial(logit)	4	−665.40	1338.86	1.96	0.37	0.16

### 3.1. Clutch size

**Figure 3.**
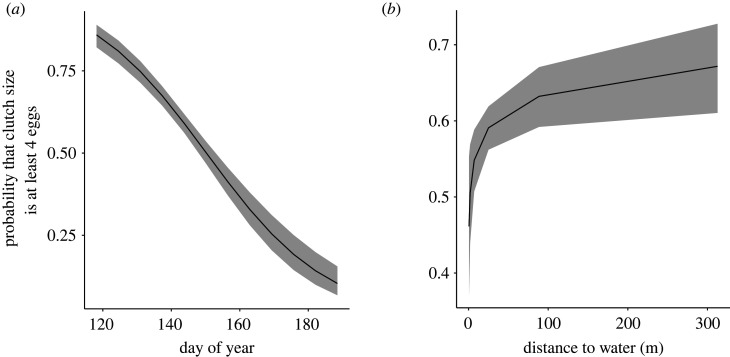
Clutch size of red-winged blackbirds decreases as (*a*) the breeding season progressed (day of year 120 = 30 April) and increases with increased distance to water (*b*). Clutch size was modelled as a bivariate factor with clutches of up to 3 eggs scored as a 0 and clutches of 4 or more eggs scored as a 1. The lines illustrate the model-predicted relationship, and shaded area illustrates 95% confidence interval. Model predictions are derived by specifying levels of the predictor variables in each figure (x-axis spans range of the data), while holding all other continuous fixed effects in the top-ranked model constant at their mean value. See main text for statistical support.

A single model best represented the relationship of the fixed effects on clutch size (table 5). Clutch size declined as the season progressed (*N* = 1293 nests, *β* = −0.72 ± 0.07, *z* = −11.01, *p* < 0.001) and increased as the distance to open water increased (*N* = 1293 nests, *β* = 0.17 ± 0.06, *z* = 2.902, *p* = 0.004; table 6 and figure 3).

**Table 6.** Fixed effect estimates (with standard error) resulting from full model averaging across all best-performing models (ΔAICc < 2) to explain variation in nest success metrics in red-winged blackbirds. Random effect intercepts were used to account for non-independence of groups. Random effects are included in models where the intra-class correlation coefficient (ICC) suggests that observations within the random variables may be correlated. The random intercept variance standard deviation (s.d.) and intra-class ICC is provided for the top-ranked model based on AICc. See main text for details of the global model. Significant effects (*p* < 0.01) are indicated in bold, and trends (0.01 < *p* < 0.05) are in italics.

fixed effects^a^				random effects		
effect	estimate (s.e.)	Z score	*p*-value	effect	random intercept variance (s.d.)	ICC
clutch size^b^						
intercept	0.37 (0.06)	6.112	**<0**.**001**	*fixed effects only*		
day of year^c^	−0.72 (0.07)	−11.013	**<0**.**001**			
distance to water^d^	0.17 (0.06)	2.902	**0**.**004**			
egg mass (g)						
intercept	15.42 (0.11)	134.815	**<0**.**001**	nest ID	2.82	0.75
incubation day^e^	−0.99 (0.11)	9.239	**<0**.**001**			
day of year^c^	0.34 (0.11)	3.038	**0**.**002**			
number of hatchlings (all locations)						
intercept	0.64 (0.24)	2.640	**0**.**008**	year	0.31	0.03
day of year^c^	−0.16 (0.03)	4.73	**<0**.**001**	region	0.39	0.04
nest height (low)^f^	−0.24 (0.07)	3.527	**<0**.**001**			
* distance to water*^d^	0.1 (0.05)	1.986	*0*.*047*			
distance to water: nest height (low)^d,f^	0.08 (0.07)	1.119	0.263			
nest density	−0.01 (0.03)	0.433	0.665			
distance to edge^g^	−0.03 (0.04)	0.714	0.475			
nest density: distance to edge^g^	−0.02 (0.04)	0.641	0.521			
PCB class	0 (0.02)	0.163	0.871			
number of hatchlings (no predated nests)						
intercept	1.7 (0.3)	5.636	**<0**.**001**	year	0.44	0.05
nest height (low)^f^	−0.45 (0.09)	4.712	**<0**.**001**	region	0.46	0.06
distance to water^d^	0.02 (0.07)	0.329	0.742			
distance to water: nest height (low)^b,f^	0.08 (0.09)	0.832	0.406			
day of year^c^	−0.03 (0.04)	0.584	0.559			
PCB class	0 (0.03)	0.113	0.910			
number of hatchlings (no predated/flooded nests)						
intercept	1.7 (0.16)	10.411	**<0**.**001**	year	0.20	0.01
day of year^c^	−0.07 (0.06)	1.184	0.236	region	0.26	0.02
distance to water^d^	0.02 (0.04)	0.442	0.658			
PCB class	0.01 (0.04)	0.224	0.823			
nestling mass (g)						
intercept	29.96 (0.25)	118.727	**<0**.**001**	nest ID	2.33	0.17
nestling age^h^	1.92 (0.17)	11.133	**<0**.**001**			
PCB class	0.5 (0.19)	2.695	**0**.**007**			
nest height (low)^f^	0.16 (0.35)	0.446	0.656			
nest density	−0.47 (0.18)	2.614	**0**.**009**			
distance to edge^g^	−0.5 (0.19)	2.659	**0**.**008**			
nest density: distance to edge^g^	−0.25 (0.21)	1.228	0.220			
day of year^c^	0.02 (0.1)	0.208	0.835			
fledging success (all locations)						
intercept	1.02 (0.2)	5.183	**<0**.**001**	year	0.38	0.04
nest height (low)^f^	−0.06 (0.09)	0.677	0.498	region	0.17	0.01
distance to water^d^	0.07 (0.06)	1.029	0.303			
*nest density*	0.12 (0.05)	2.517	*0*.*012*			
distance to edge^f^	−0.05 (0.05)	0.967	0.333			
distance to water: nest height (low)^d,f^	−0.26 (0.09)	2.890	**0**.**004**			
nest density: distance to edge^g^	0.04 (0.05)	0.849	0.396			
PCB class	−0.03 (0.06)	0.430	0.667			
day of year^c^	0.01 (0.03)	0.420	0.674			
fledging success (no predated nests)						
intercept	1.99 (0.34)	5.752	**<0**.**001**	year	0.64	0.11
distance to water^d^	−0.01 (0.04)	0.264	0.792	region	0.41	0.04
PCB class	−0.01 (0.05)	0.181	0.856			
day of year^c^	0 (0.03)	0.134	0.893			
fledging success (no predated/flooded nests)						
intercept	2.13 (0.3)	7.090	**<0**.**001**	year	0.56	0.08
distance to water^d^	−0.04 (0.06)	0.590	0.555	region	0.34	0.03
PCB class	0 (0.05)	0.096	0.923			

^a^Parameter estimates are comparable across fixed effects because they were scaled (mean of 0 and s.d. of 1).

^b^Clutch size was treated as a binomial variable for analyses, with clutches less than or equal to 3 coded as 0 and greater than or equal to 4 coded as 1.

^c^Day of year is the calendar day of the onset of incubation (with 1 May = 121).

^d^Distance to water is the shortest distance to open water in wetlands or the river.

^e^Incubation day is the estimated day of the approximately 14-day incubation period when eggs were weighed, included as a covariate to control for water loss.

^f^Nest height is categorical, with nests described as low (less than or equal to 0.6 m) or high (greater than 0.6 m).

^g^Distance to edge is the shortest distance to woods or human structures.

^h^Nestling age is the estimated age of the nestlings when they were weighed, included as a covariate to control for growth over nestling development.

### 3.2. Egg mass

**Figure 4.**
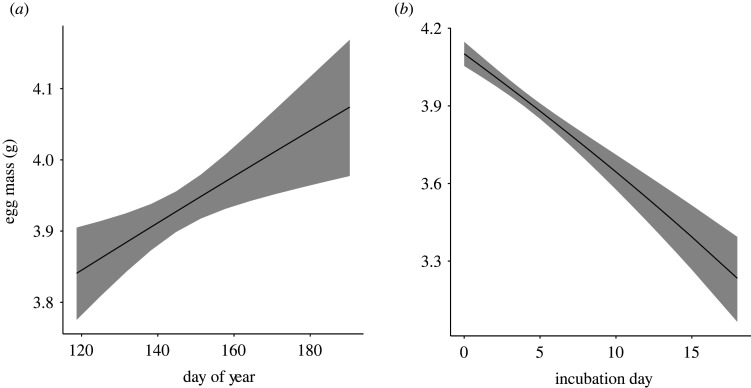
Mass of red-winged blackbird eggs increased as (*a*) the breeding season progressed (day of year 120 = 30 April) and decreases with the day of incubation (*b*). The line illustrates the model-predicted relationship, and shaded area illustrates 95% confidence interval. Model predictions were based on the highest-ranking model as indicated by the lowest AICc of all candidate models and included both day of year and day of incubation. See main text for statistical support.

As with clutch size, a single model best represented the relationship of the fixed effects on egg mass (table 5). Egg mass increased as the breeding season progressed (*β* = 0.34 ± 0.11, z = 3.04, *p* = 0.002; table 6 and figure 4*a*), but declined over the course of incubation, as expected due to water loss (*N* = 2308 eggs from 685 nests, *β* = −0.99 ± 0.11, z = 9.24, *p* < 0.001; table 6 and figure 4*b*).

### 3.3. Hatching success

**Figure 5.**
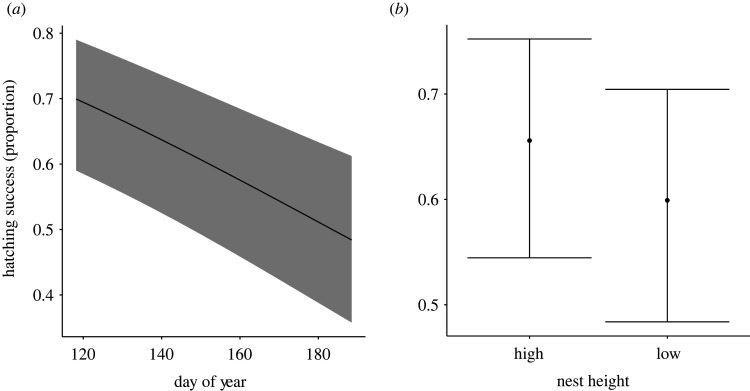
Model-predicted relationships between hatching success in red-winged blackbird nests (proportion of eggs that hatched) and nest characteristics with all nests included in the analyses. Hatching success (*a*) decreased significantly over the breeding season. Hatching success (*b*) was also lower in nests that were built within 0.5 m of the ground surface. Nests higher than 0.5 m displayed greater hatching success. Lines illustrate model-predicted linear relationships, and shaded areas illustrate 95% confidence intervals. Model predictions are derived by specifying varying levels of the fixed effects illustrated in the figure, while holding all other fixed effects in the top-ranked model constant. Continuous variables were set at their mean value, and the categorical factor (nest height) was specified as the proportion of nests that were low (i.e. nests less than or equal to 0.5 m off the ground). See main text for statistical support.

In analysis of the full dataset (*N* = 1293 nests), which retained data from all nests, including those that failed due to predation or flooding during the incubation stage, hatching success declined as the breeding season progressed (*β* = −0.16 ± 0.03, *z* = 4.73, *p* < 0.001, table 6 and figure 5*a*). Hatching success was also lower in nests that were below 0.5 m from the ground (*β* = −0.24 ± 0.07, *z* = 3.50, *p* < 0.001, table 6 and figure 5*b*). There is a trend for the number of hatchlings to increase with distance to water (*β* = 0.10 ± 0.05, *z* = 1.99, *p* = 0.047, table 6). No other fixed effects or interactions retained in models ranked within 2 AICc of the top-ranked model predicted hatching success (table 5).

**Figure 6.**
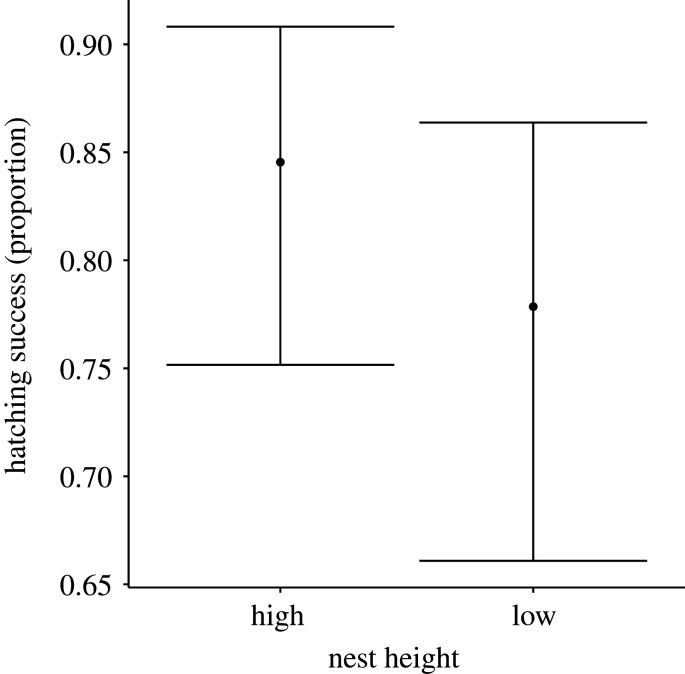
Model-predicted relationships between hatching success in red-winged blackbird nests (proportion of eggs that hatched) and nest characteristics when known predated nests were removed from the analysis. When compared with analyses that included all nests, hatching success was still lower in nests that were built within 0.5 m of the ground surface. Nests higher than 0.5 m displayed greater hatching success. Model predictions are derived by specifying varying levels of the fixed effects illustrated in the figure, while holding all other fixed effects in the top-ranked model constant. Continuous variables were set at their mean value, and the categorical factor (nest height) was specified as the proportion of nests that were low (i.e. nests less than or equal to 0.5 m off the ground). See main text for statistical support.

When analyses were repeated with nests that failed due to predation during the incubation stage excluded from analyses (*N* = 1019 nests retained, 274 depredated nests excluded), results were similar to those from the full dataset. Hatching success was lower in low nests relative to higher nests (*β* = −0.45 ± 0.09, *z* = 4.71, *p* < 0.001, table 6 and figure 6).

When the analysis was repeated with both nests that were depredated as well as nests that failed due to flooding during the incubation stage excluded (*N* = 971, 48 flooded and 275 depredated nests excluded), results differed somewhat from those of the analysis that only excluded depredated nests. Hatching success no longer differed between low and high nests, as is expected if lower nests are more likely to fail due to flooding (table 6).

### 3.4. Nestling mass

**Figure 7.**
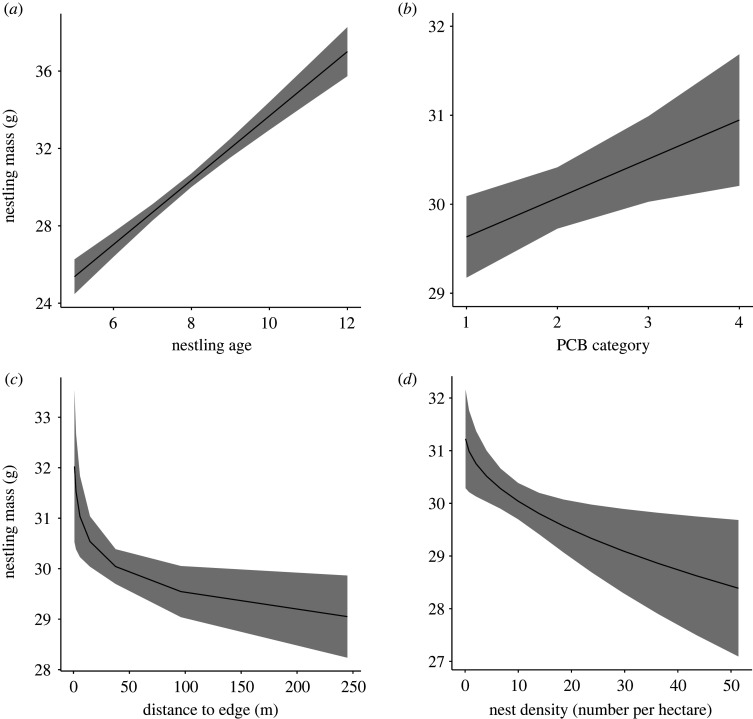
Mass of red-winged blackbird nestlings increased with nestling age (*a*) and local PCB level (*b*) and decreased with distance to edges (*c*) and nest density (*d*). The lines illustrate model-predicted relationships, and shaded areas illustrate 95% confidence interval. Model predictions are derived by specifying levels of the predictor variables in each figure (*x*-axis spans range of the data), while holding all other continuous fixed effects in the top-ranked model constant at their mean value. The categorical factor (nest height) was specified as the proportion of nests that were low (i.e. nests less than or equal to 0.5 m off the ground). See main text for statistical support.

As expected, nestling mass increased with nestling age (*N* = 1446 nestlings from 512 nests, *β* = 1.90 ± 0.17, z = 10.96, *p* < 0.001, table 6 and figure 7*a*). Nestling mass also increased with PCB exposure (*β* = 0.50 ± 0.19, z = 2.70, *p* = 0.007, table 6 and figure 7*b*), but decreased with distance to edge (wooded or human structure: *β* = −0.50 ± 0.19, *z* = 2.66, *p* = 0.008, table 6 and figure 7*c*) and nest density (*β* = −0.47 ± 0.18, *z* = 2.61, *p* = 0.009, table 6 and figure 7*d*). No other fixed effects or interactions retained in models ranked within 2 AICc of the top-ranked model predicted nestling mass (table 5).

### 3.5. Fledging success

**Figure 8.**
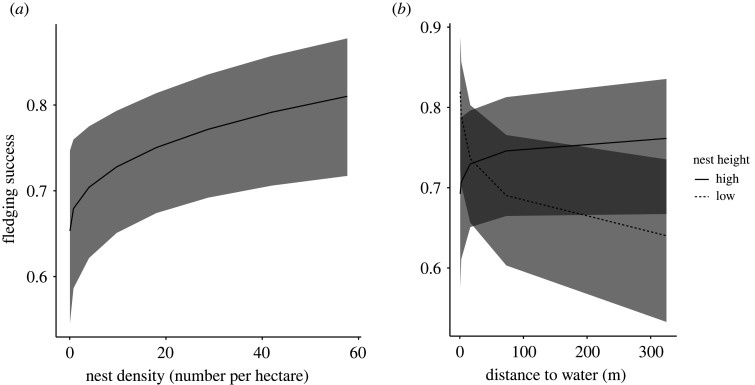
Model-predicted relationships displaying the increase in fledging success with increased nest density (*a*). Model-predicted relationships between fledging success in red-winged blackbird nests (proportion of nestlings that fledged), nest height and distance to open water or wetlands (*b*). In nests built within 0.5 m of the ground, fledging success decreased with increasing distance to open water or wetlands. By contrast, there was no significant change in fledging success in nests built higher than 0.5 m from the ground. Lines illustrate model-predicted linear relationships but might appear nonlinear due to back-transformation of data. Shaded areas illustrate 95% confidence intervals. Model predictions are derived by specifying levels of the predictor variables in each figure (*x*-axis spans range of the data), while holding all other continuous fixed effects in the top-ranked model constant at their mean value. See main text for statistical support.

Fledging success was best predicted by the interaction of distance to open water and nest height (table 6 and figure 8). In the full dataset (*N* = 922 nests), which retained data from all nests where at least one nestling hatched, including those that failed due to predation or flooding during the nestling stage, the relationship between fledging success and distance to open water depended on nest height (*β* = −0.26 ± 0.09, *z* = 2.89, *p* = 0.004). Specifically, in nests that were closer to the ground, fledging success decreased with increased distance to water (figure 8). Results also show a trend towards fledging success increasing with nest density (*β* = 0.12 ± 0.05, *z* = 2.52, *p* = 0.012, table 6). No other fixed effects or interactions retained in models ranked within 2 AICc of the top-ranked model predicted fledging success (table 5).

When analyses were restricted to exclude nests that failed during the nestling phase due to predation (*N* = 778 nests retained, 144 depredated nests excluded) or failed due to predation and flooding (*N* = 767 nests retained, 11 flooded nests excluded), there were no significant predictors of fledging success (table 6).

### 3.6. Daily nest survival

**Figure 9.**
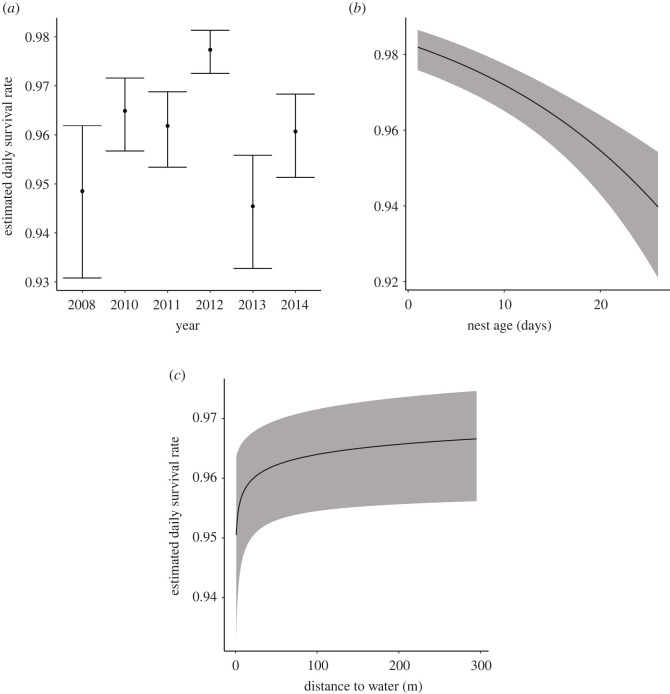
Model-predicted relationships displaying estimated daily survival in relation to year, nest age and distance to open water. Average estimated daily survival was best represented by 2014 (*a*). Estimated daily survival decreased with nest age (*b*) but increased with distance to water (*c*). Shaded areas illustrate 95% confidence intervals. Model predictions are derived by specifying levels of the predictor variables in each figure (x-axis spans range of the data), while holding all other continuous fixed effects in the top-ranked model constant at their mean value and using 2014 as the base year. See main text for statistical support.

Daily nest survival was best predicted by the model that included the variables year, nest age, day of year and distance to open water (table 7). The average rate of daily nest survival for all years is 0.96. This survival rate was best represented by daily nest survival in 2014, which was used as the base year to present model results for all other significant parameters (figure 9). The confidence intervals of both nest age and distance to water did not include zero, indicating strong support for their potential effects on daily nest survival (table 8). As expected, daily nest survival decreased with nest age (*β* = −0.05 ± 0.01, table 8). Conversely, daily nest survival increased with increased distance to water (*β* = 0.17 ± 0.08, table 8). Three additional models were within 2 AICc of the top-ranked model predicting daily nest survival (table 7). The second and third ranked models were variants of the top-ranked model. The fourth ranked model also included PCB and the interaction of PCB and distance to water; however, confidence intervals of both parameters included zero suggesting a lack of support for their effects.

**Table 7.** Summary statistics of top-ranked candidate models that compare daily nest survival rates of red-winged blackbird with habitat and nest characteristics. Models were ranked by AICc. Years were included as a dummy variable to assess effects between years. Data evaluated were collected from marshes in 2008–2014 in various regions along the upper Hudson River and Mohawk River.

model	number of parameters	AICc	delta	model likelihood	AICwt
year + nest age + day of year + distance to water	9	3103.310	0.00	1.000	0.366
year + nest age + distance to water	8	3101.357	0.05	0.976	0.358
year + nest age + day of year	8	3103.264	1.95	0.377	0.138
year + nest age + day of year + (PCB class × distance to water)	11	3103.270	1.96	0.375	0.138

**Table 8.** Effect estimates (with standard error) resulting from the best-performing model to explain variation in daily nest survival rates in red-winged blackbirds. Years were included as a dummy variable to assess effects between years. All analyses were conducted using a logistic exposure model in RMark. Parameters estimates with confidence intervals that do not include zero are indicated in bold.

effect	*β* estimate	s.e.	95% lower confidence limit	95% upper confidence limit
intercept	4.34	0.60	**3**.**15**	**5**.**52**
year: 2010	0.36	0.20	−0.02	0.75
year: 2011	0.29	0.19	−0.09	0.66
year: 2012	0.81	0.19	**0**.**45**	**1**.**18**
year: 2013	−0.09	0.19	−0.47	0.29
year: 2014	0.25	0.20	−0.14	0.63
nest age	−0.05	0.01	**−0**.**07**	**−0**.**03**
day of year^a^	−0.01	3.76E-03	−0.01	1.93E-03
distance to water^b^	0.17	0.08	**0**.**00**	**0**.**33**

^a^Day of year is the calendar day of the onset of incubation (with 1 May = 121).

^b^Distance to water is the shortest distance to open water in wetlands or the river.

## 4. Discussion

We found little evidence that characteristics associated with the human-dominated landscapes in the region were negatively correlated with nesting productivity of RWBLs. There were no edge-related reductions of nesting success, a reliable index of fitness [90], even for the few nests located immediately adjacent to wooded edges where predator communities may be different [69]. By contrast, we found that nestling mass was higher when nests were closer to wooded edges or buildings. As expected, nestling mass was also greater in areas with lower nest density, an effect that has been observed in other species nesting in human-modified landscapes [91]. Additionally, we found no evidence that exposure to PCBs was negatively correlated with nesting success of RWBLs in the region. In fact, PCB exposure was positively related to nestling mass; however, it is likely that the increase in nestling mass was not a direct result of PCB exposure, but instead the result of an unmeasured variable correlated with areas of higher PCB exposure. Nest height was related to hatching success because lower nests were more likely to be flooded during the incubation phase. This was evident with the continued negative effect of nest height in models that analysed hatching success in nests not affected by predation, which might also be affected by nest height. Nesting success declined with date within the season, a reflection of decreasing clutch size and hatching success, but egg mass increased.

### 4.1. Edge effects

The relative lack of strong effects of distances to open water and wooded edges (or human structures) on nesting productivity in this study is generally consistent with the other studies that have examined edge effects in RWBLs [21,65]. Only nestling mass was found to have a significant relationship with distance to wooded edges. These results are consistent with Whittingham & Robertson [92] who found that parental provisioning was higher when parents foraged off-territory in woodland habitat. The increase in clutch size and daily survival rate with increased distance to water was not consistent with predictions based on protection provided by deeper, open water. Picman *et al*. [65] found that water depth, not distance to edge, had a significant effect on predation rates in marshes, which they attributed to deeper water providing protection against mammalian nest predators. A similar mechanism may be responsible for the observed decrease in fledging success in nests built close to the ground when associated with increased distance to water. We did not, however, measure water depth under nests during our study and therefore do not have strong tests of this hypothesis. Our results also differ from those of Vierling [29], who found that nesting sites close to human habitation had significantly lower nesting success than those in other landscape contexts. A review of the many studies of RWBL nesting success [42] found no consistent effects of open water and water depth on nesting success of RWBLs. Higher predation rates in nests adjacent to forests might reflect vulnerability to forest-based predators such as some snakes and raccoons (*Procyon lotor*), which rarely visit deep water [93], but we found no consistent negative effects of distance to woody vegetation or buildings. Our results add to the growing number of studies that show few obvious edge effects on nesting success of birds in wetlands and grassy habitats [11].

### 4.2. Exposure to PCBs

Nesting success of RWBLs was not reduced in marshes with relatively high PCB concentrations compared with marshes with low PCB concentrations and with presumably limited or no PCB exposure. Nestling mass increased significantly with likely exposure to PCBs; given the lack of known mechanisms by which PCBs would increase nestling mass, it is possible that this reflects differences in the quality of food available in marshes. Although results were based on an index of potential PCB exposure rather than measured PCB concentrations in eggs or nestlings, results are consistent with other studies that have shown no effects of PCB exposure on nesting success [6,33,51,52,56,94]. RWBL foraging patterns are highly variable and increase the uncertainty of PCB exposure to marsh-nesting RWBLs that frequently forage in upland locations [95]. Adult RWBL are known to feed their young predatory aquatic odonates [35,37–39] that are assumed to bioaccumulate toxins; feeding patterns, however, are highly variable. Although RWBLs are considered to be a species of intermediate sensitivity to PCBs [60], at least some eggs contain high concentrations of PCBs in the region (electronic supplementary material, figure S1; [81]). Additional studies that examine nesting success in conjunction with egg analysis would provide additional support for the lack of potential effects of PCBs on RWBL nest success [35,37–39]. The lack of effects of likely PCB exposure on nesting success does not preclude possible adverse effects associated with other environmental contaminants, but it does show the continued need for field studies to understand the relative effects of toxins compared with other factors [96] including anthropogenic factors such as vehicle or structure collisions, wetland alteration, predation, or other myriad human effects [97].

### 4.3. Other factors

#### 4.3.1. Nest height

Our data suggest that the primary risk associated with nesting low over the water is associated with flooding, which was primarily observed where water-level management occasionally led to spikes in water levels during the nesting season (e.g. Lions Park on the Mohawk River). Overall, only 4% of the nests were flooded. In fact, in nests closer to the ground, fledging success was higher in areas closer to water. Any increases in flooding frequency during the nesting season brought about by changing climates (e.g. higher frequency of severe summer rainstorms) [98,99] or by altered hydrological cycles caused by changes in drainage or water-level management in impoundments could increase losses to flooding during the breeding season.

#### 4.3.2. Within-season variation

A decline in clutch size, hatching success and daily survival rate as the season progresses has been widely documented in birds and other vertebrates [70,100] and has been documented specifically in RWBLs [35,64]. Interestingly, the smaller late-season clutches had eggs that were, on average, larger than early season eggs. This pattern has been widely documented in vertebrates [63] and might represent a way in which RWBLs compensate for smaller clutches by laying eggs with more supplies to combat declining food supplies as marshes drain or to give their fledglings a greater chance of surviving in the face of competition with fledglings that left the nest earlier in the breeding season. Alternatively, it might be a response to increasing day length during the breeding season, providing more time for follicular growth during egg development [101].

#### 4.3.3. Nesting density

Changing population densities of RWBLs should have contrasting effects on nest predation rates and on the growth and development of young. Nesting success should increase if mobbing effectively deters nest predators [45], but productivity from nests that escape predation should be lower if there are negative density-dependent reductions in food within marshes or in the nearby upland habitats [42]. If RWBLs are forced to nest at increasingly high population densities as a result of decreasing habitat availability, then increasing human land use could lead to lower nest predation rates, but reduced productivity from nests that survive. Alternatively, decreased regional populations may lead to decreased nesting density within marshes potentially leading to increased body condition of nestlings within surviving nests, but also increased nest predation rates. Our results support these predicted effects as we documented declining nestling mass in areas of higher density. Our results suggest that, although not significant at *α* = 0.01, increased nest density may have increased productivity through a reduction in potential predation. Avian nest predators can be deterred by mobbing behaviours [42]. In RWBLs, this is especially evident when defending against marsh wrens (*Cistothorus palustris*), which are prominent RWBL nest predators [43] and were present in most marshes (unpublished data). Although probably not as effective, mobbing behaviour has also been observed for mammalian nest predators such as mink [102] and raccoons [103], both of which are common in and around the Hudson River and its tributaries.

#### 4.3.4. Possible additional factors

Other factors that have been listed as possible causes of reproductive failure that we have not studied here include other toxins such as methyl mercury and pesticides; reduced insect populations; proliferation of invasive species; decreasing marsh size; loss of upland nesting habitat; climate change; and direct mortality to pest control efforts [1]. The other factors listed can be related to human disturbance. In addition, suburban and rural landscapes have been shown to act as population sinks for RWBL [29] suggesting that measured nesting characteristics may have variable effects with differing levels of human disturbance. It is notable that because all marshes in the study area were located in rural locations with moderate levels of human influence, there is no measured range of human influence or reference areas to directly assess potential effects of increased or decreased human disturbance. Our marshes are relatively free of invasive species and there were no obvious differences in nesting success in the few nests we found in invasive plants (unpublished data). We had no data from upland habitats because our study was designed originally to focus on nests that would be exposed to PCBs in the floodplain. For these variables, our analysis offers no insights.

## 5. Conclusion

Several variables including nest height and distance to open water or edges, both alone and in combination, had statistically significant effects on RWBL nesting success. However, few of the effects we documented were associated with large decreases in nesting success. Also, PCBs were not found to be negatively correlated with nesting success in the upper Hudson River. These results suggest that the long-term population declines of RWBLs in this region [2] may be caused by other factors such as outright loss of marsh habitat [22], climate change including an increase in summer flooding [99,104–106], and intensification of agriculture, which can include loss of landscape heterogeneity [12] and earlier harvesting of hayfields where they also nest [13,28,36,107]. Alternatively, these declines could be caused by factors outside the breeding season, including killing of birds at roosting or feeding aggregation sites.

## Data Availability

The datasets supporting this are openly available at the Dryad Digital Repository: https://doi.org/10.5061/dryad.ht76hdrhx [108]. Related software is published at Zenodo: https://doi.org/10.5281/zenodo.6323697 [109]. The data are provided in electronic supplementary material [110].
